# Mild Gestational Hyperglycemia in Rat Induces Fetal Overgrowth and Modulates Placental Growth Factors and Nutrient Transporters Expression

**DOI:** 10.1371/journal.pone.0064251

**Published:** 2013-05-14

**Authors:** Ouma Cisse, Isabelle Fajardy, Anne Dickes-Coopman, Emmanuelle Moitrot, Valérie Montel, Sylvie Deloof, Jean Rousseaux, Didier Vieau, Christine Laborie

**Affiliations:** 1 Unité Environnement Périnatal et Croissance, EA 4489, Université Lille Nord de France, Lille, France; 2 Pôle de Biochimie et Biologie Moléculaire, Centre de Biologie et de Pathologie, Lille, France; Pennington Biomedical Research Center/LSU, United States of America

## Abstract

Mild gestational hyperglycemia is often associated with fetal overgrowth that can predispose the offspring to metabolic diseases later in life. We hypothesized that unfavorable intrauterine environment may compromise the development of placenta and contribute to fetal overgrowth. Therefore, we developed a rat model and investigated the effects of maternal dysglycemia on fetal growth and placental gene expression. Female rats were treated with single injection of nicotinamide plus streptozotocin (N-STZ) 1-week before mating and were studied at gestational day 21. N-STZ pregnant females displayed impaired glucose tolerance that is associated with a lower insulin secretion. Moderate hyperglycemia induced fetal overgrowth in 40% of newborns, from pregnancies with 10 to 14 pups. The incidence of macrosomia was less than 5% in the N-STZ pregnancies when the litter size exceeds 15 newborns. We found that placental mass and the labyrinthine layer were increased in macrosomic placentas. The expression of genes involved in placental development and nutrient transfer was down regulated in the N-STZ placentas of macrosomic and normosomic pups from pregnancies with 10 to 14 ones. However, we observed that lipoprotein lipase 1 (LPL1) gene expression was significantly increased in the N-STZ placentas of macrosomic pups. In pregnancies with 15 pups or more, the expression of IGFs and glucose transporter genes was also modulated in the control placentas with no additional effect in the N-STZ ones. These data suggest that placental gene expression is modulated by gestational conditions that might disrupt the fetal growth. We described here a new model of maternal glucose intolerance that results in fetal overgrowth. We proposed that over-expression of LPL1 in the placenta may contribute to the increased fetal growth in the N-STZ pregnancies. N-STZ model offers the opportunity to determinate whether these neonatal outcomes may contribute to developmental programming of metabolic diseases in adulthood.

## Introduction

The intrauterine metabolic environment in which the fetus develops constitutes to date, a crucial determinant of fetal programming of chronic diseases in adulthood [1], [2]. Exposition *in utero* to maternal diabetes represents a detrimental environment which despite advances in treatment remains associated with maternal and neonatal complications [3]. Gestational dysfunctions related to maternal diabetes are not restricted to women with overt diabetes diagnosed before gestation, but are also observed in gestational diabetes (GDM), a condition defined as glucose intolerance with onset or first recognition during pregnancy [4]. Furthermore, infants born from mothers with diabetes or GDM have increased predisposition to obesity and T2DM in adulthood [5], [6]. Recently, it has been reported that mild gestational hyperglycemia (MGH) also induced several perinatal anomalies [7], [8]. In particular, the incidence of large-for-gestational-age (LGA) or macrosomic infants is increased in pregnancies complicated with MGH [7], [9].

Fetal growth is the result of the balance between the nutrient demand of the fetus and the maternal nutrient supply. Availability of nutrients in turn, depends on the maternal metabolism, utero-placental blood flow and placental transfer efficiency. However, recent studies demonstrate that the functions of the placenta are not limited to maternal-fetal exchange. In response to metabolic challenges, the placenta may adapt its functional capacity by modifying its morphology and/or nutrient transporter activity and, thereby, could contribute to the developmental programming of disease susceptibility of the offspring [10]–[12]. To date, the molecular mechanisms underlying the modifications of fetal growth in the context of pregnancies complicated with moderate hyperglycemia have not been investigated. As in diabetic pregnancies, the placenta from women with MGH showed vascular dysfunctions and major tissue damage that could affect the placental function and thus compromise fetal development [13], [14].

To our knowledge, there is no relevant model described in the literature for the study of the placenta in relation to fetal growth. Most of the experimental approaches used to generate mild or severe diabetes during pregnancy have relied on the destruction of pancreatic β-cells through the administration of drugs such as streptozotocin (STZ) during neonatal period, before mating or during pregnancy [15], [16]. However, experimental paradigms that have used STZ alone generate a more severe maternal diabetic state that relates to T1DM and results in intrauterine growth retardation rather than fetal overgrowth. In order to obtain an experimental model of mild hyperglycemia during the gestation, we have injected animals with nicotinamide, in addition to streptozotocin. Nicotinamide, a water soluble B3 vitamin has been used in several species to protect the pancreatic β cells against the toxic effect of STZ [17]–[19]. Nicotinamide inhibits the activation of nuclear poly (ADP-ribose) polymerase and prevents nicotinamide adenine dinucleotide (NAD) depletion and β cell death induced by STZ [20, 21). STZ rats previously administered with nicotinamide exhibited moderate hyperglycemia as the consequence of partial reduction of beta cells mass and impaired insulin secretion [17], [19]. We used the N-STZ model to determinate the effects of the moderate hyperglycemia of the mother on the fetal growth and to test the hypothesis that placental dysfunctions might be linked to abnormal growth of the fetus. Our results suggest that impaired glucose tolerance in mother induces fetal overgrowth and modulates placental expression of genes involved in nutrients transport and placental development. We observed that litter size influences the gene expression and the placental phenotype. Our results are in accordance with the hypothesis of “placental plasticity” that reflects the ability of placenta to adapt its morphology and function to maximize fetal growth and viability at birth.

## Research Design and Methods

### Ethics Statement

Animal use accreditation by the French Ministry of Agriculture (No 04860) has been granted to our laboratory for experimentation with rats. All animal experiments were conducted in accordance with the European Communication Council Directive of November 24, 1986 (86/609/EEC) and approved by the French Departmental Direction of Veterinary Services Committee (DDSV/59-009228). All surgery was performed under ketamine anesthesia, and all efforts were made to minimize suffering.

### Animals and Experimental Protocol

Female rats of the Wistar inbred strain (8–9 weeks old, Janvier, Le Genest Saint Isle, France) were housed five per cage and accommodated to the animal facility for one week before any experimentation. There were maintained upon controlled 12/12 hours light/dark cycles and temperature (22±2C) and fed *ad libitum* with a standard chow diet (16% protein, 3% fat and 60% carbohydrate, SAFE, Augy, France). Females were randomly assigned to either control (C) or nicotinamide-streptozotocin (N-STZ) groups and were housed individually. N-STZ females received an i.p injection of nicotinamide (230 mg/kg body weight diluted in NaCl 0.9%, Sigma-Aldrich Corp., St Louis, MO, USA) following 15 min after by an i.p injection of streptozotocin (64 mg/kg body weight diluted in 0.1 m/l citrate buffer, pH 4.5, Sigma). Administration of nicotinamide alone at dose of 230 mg/kg was without effect on body weight and plasma insulin and glucose levels [22]. Control females were injected with the same volume of citrate buffer. N-STZ females (n = 6 females) whose non-fasting glucose values exceeded 13.9 mmol/l were diabetics. They required insulin injections and were excluded from further analysis. The females were set up for mating no earlier than 7 days after the last N-STZ injection. On the day the vaginal plug was detected represents the gestational day 0 (GD0). Pregnant females were housed individually with *ad libitum* access to standard chow. Body weight and non fasting glucose values were measured every week during the pregnancy. Oral glucose tolerance test (OGTT) was performed in all females just before mating, and in late gestation at GD19.

### Glucose tolerance test

OGTT was performed on C and N-STZ virgins and pregnant females, after an overnight fast. Conscious unrestrained animals were administered *per os* with glucose (2 g/kg body weight), and blood samples were collected from the tail vein at 0, 30, 60, 90 and 120 min for glucose determination (ACCU-CHEK Peforma Glucometer, Roche Diagnostics, Mannheim, Germany). At 0 and 30 min, a blood sample was collected in EDTA rinsed tube and plasma was frozen at −20°C for subsequent insulin determination.

### Collection of blood and tissue samples

Dams were euthanized on GD21 by intra peritoneal injection of ketamine (125 mg/kg body weight) and maternal blood was collected by cardiac puncture. After laparotomy, both horns of the uterus were excised and the fetuses were removed from the uterus with their respective intact placenta and weighed. For each litter, the placentas were classified according to sex and birth weight and then were randomly distributed for morphological and molecular analyses. Fetal blood was obtained by an axillary incision and plasma samples from each offspring were pooled within the same litter according to the sex. Plasma insulin was determined using Ultra Sensitive Elisa kit (DRG International, Inc, USA). Plasma non esterified fatty acids (NEFA) and triglycerides (TG) concentrations were measured using enzymatic assay kits (Wako Chemicals, Neuss, Germany and Laboratoires SOBIODA, Montbonnot, France, respectively).

### Quantitative real-time PCR analysis of placental gene expression

We selected for each litter the female and male placentas (ratio: 1) so as to obtain “normosomic (n = 4) and macrosomic (n = 4) samples according to the criteria of birth weight that were detailed in the Results section. Placentas were washed in NaCl 0.9% and rapidly transferred in RNA later (Qiagen, Inc, Valencia, CA, USA) for 24 h and then frozen at −80°C. Placental biopsies (30 mg) were prepared from the labyrinthine layer on both sides of the insertion zone of the chorionic plate. RNAs were extracted from the samples using the Qiagen RNeasy Mini Kit (QIAGEN, Crawley,UK) and their concentration was determined using a NanoDrop spectrophotometer. The integrity of RNA was evaluated by gel electrophoresis. For each sample, 1 µg of total RNA was reverse transcribed to cDNA using the ThermoScript RT Kit (Invitrogen Life Technologies, Carlsbad, CA, USA). All cDNA samples were analyzed in duplicate and reaction was carried out with QuantiTect SYBR Green Master mix (Qiagen). Normalization was done to 3 housekeeping genes cDNA in the same sample; cyclophilin A (Cyclo A), β2 microglobulin (β2M) and HPRT, whose expression remained constant across groups. Primers were designed using primer quest software (Primer-Blast, http://www.ancbi.nlm.nih.gov/tools/primer-blast/) and were positioned to span exon-exon junctions to exclude amplification of potentially contaminating genomic DNA. Primer pairs used in this study (Eurogentec, Inc, San Diego, CA, USA) are listed in Table 1. Real-time RT-PCR experiments were performed using LightCycler 2700 System (Roche Biochemicals, Meylan, France). Analysis of transcript level was carried out using first determination of the threshold cycle Ct for each reaction corrected by the efficiency as previously described [23]. For each litter, individual data from females and males were averaged, so as the “n = 1” represented the value obtained for the entire litter.

**Table 1:** Primers sequences used for quantitative real-time PCR assays.

Gene symbol	Primer sequence		
	Accession Nb	Forward	Reverse
**Cyclophilin A (Ppia)**	NM_017101	ATTCATGTGCCAGGGTGGT	GATGCCAGGACCTGTATGCT
**β2 microglobulin (β2M)**	NM_012512	ACCTGGGACCGAGACATGTA	GAAGATGGTGTGCTCAATTGC
**Hypoxanthin guanine phosphoribosyl transferase (HPRT)**	NM_012583	GTGTCATCAGCGAAAGTGGA	ATGGCCACAGGACTAGAACG
**Insulin receptor (INSR)**	NM_017071	AACAACCCTGGCAGCTGAACT	TTCCAAGGTCTCTCTCGAA
**Insulin Related Substrate 1 (IRS1)**	NM_0129969	ACAGAGAGTGGACCCCAATG	ATGCTGCTACTGCTGCAAGA
**Insulin Related Substrate 2 (IRS2)**	XM_0011076309	CTCTTTGCCCCGCTCTTAC	GCTCCAGTCTCTCCTCTTCC
**Insulin Like Growth Factor 2 (IGF2)**	NM_31511	CGCGGCTTCTACTTCAGCA	GTCTCCAGGAGGGCCAAG
**Insulin Like Growth Factor Receptor 1 (IGF1-R)**	NM_052807	TTGGAGATTTTGGTATGACACG	GAGGGACTCGGGAGACATC
**Insulin Like Growth Factor Receptor 2 (IGF2-R)**	NM_012756	CCGTGTGTGCTGTGGATAAG	CACAGTCATCGCCATCAGAG
**Glucose Transporter 1 (GLUT 1)**	NM_138827	ATTAAAAAGGTGACGGGCC	CGGTGGTTCCATGTTTGATT
**Glucose Transporter 3 (GLUT 3)**	RN_98055	GACCAAGCGACGGAGATC	AGAGCTCCAGCACAGTGACC
**Glucose Transporter 4 (GLUT 4)**	NP_036883	AGGCACCCTCACTACCCTTT	ATAGCCCTTTTCCTTCCCAA
**Amino acid transporter 1 (SNAT1)**	RN_162022	CGTGACTGACGCGTGCACACAC	AGACGGGTGGCAGACAAACGC
**Amino acid transporter 2 (SNAT2)**	NM_1801090	AAGAGCTGAAGAGCCGCAGC	CGGCAAGCAAATACATCAGA
**Amino acid transporter 4 (SNAT4)**	NM_001107078	CTGTTCCCCATCCGTACTTC	GACGTTGTTGAGAGCGATGA
**Amino acid transporter (LAT1)**	NM_031341	GATGCCTGCATCTGTCTCTTA	CAATCAGCGCCAACACTTTA
**Lipoprotein lipase 1 (LPL 1)**	NM_012598	AAGGTCATCTTCTGTGTCCA	CAGCCCGACTTCTTCAGAGA
**Lipoprotein lipase endothelial (LIPG)**	NM_001012741	GCAGGATCACTGGATTGGAT	CAGCGTGTAGGTATGCAGGA
**Lipoprotein lipase hormone sensitive (LIPE)**	NM_0128591	CTACTGGGATACAGCCTCGG	AAGGCCATGTTGTCTTCTGC
**Very low density lipoprotein receptor (VLDLR)**	NM_0131552	CCCTGAAGGAGTGTGCCATATC	CAGCTGCACAGTCACATTCA
**Placental Growth Factor (PLGF)**	RN_6960	CGCTAAAGACAGCCAACATC	ATTCGCAGAGCACATCCTG
**Vascular Endothelial Growth Factor A (VEGF)**	NM_031836	CTGGAGCGTTCACTGTGAG	GCGAGTCTGTGTTTTTGCAG

### Western blot Analysis

We selected for each litters, one placenta (male or female), according to the normosomic and macrosomic criteria, to obtain four to six placenta samples per group. Placentas were snap frozen in liquid nitrogen for analysis of protein expression by Western Blotting. Proteins were extracted from whole placentas in lysis buffer containing 250 mmol/l sucrose, 10 mmol/l Hepes-Tris (pH 7.4), 1 mmol/l EDTA, 1% Triton X-100, and 1∶1000 proteases inhibitor cocktail (Sigma). Protein concentrations were determined by Lowry method using *DC* Protein assay (Bio-Rad Laboratories, Marnes-La-Coquette, France). Equivalent amounts of protein (10 μg) were submitted to a 10% SDS-PAGE gel electrophoresis, and transferred onto nitrocellulose membrane (Hybond-ECL, Amersham BioSiences, Chalfont, UK). Membranes were blocked overnight at 4°C with 5% non fat dry milk in TBS buffer containing 0.1% Tween 20, and then incubated with a rabbit polyclonal antibody anti-LPL (H-53) (1∶400, Santa Cruz Biotechnology, CA, USA) overnight at 4°C. After washing, membranes were incubated with goat anti rabbit IgG HRP conjugate (1∶2000, Bio-Rad Laboratories) for 1 h at room temperature. After washing, bands were visualized using enhanced chemiluminescence (ECL) detection reagents (Amersham BioSciences). Blots were stripped and reprobed for α-tubulin as a loading control (Abbiotech, San Diego, CA, USA). Lysates from neonatal heart served as positive controls. Values for LPL1 protein were expressed relative to α-tubulin. Each group consisted of 4 to 6 samples from independent litters.

### Placental structure analysis

Placentas (n = 2, male and female ratio: 1) from three or four independent litters were selected according to the “normosomic “and “macrosomic “criteria. Placentas were fixed in 4% (wt/vol. in phosphate buffer) paraformaldehyde and embedded in paraffin wax. Using a rocking microtome, the 6 µm vertical sections were cut; the chorionic plate providing the theoretical horizontal plane. Mid-sagittal sections were mounted on SuperFrost Plus slides and stained with hematoxylin-eosin-safran. Ten sections from each placenta and two distinct regions localized within mid-sagittal sections were quantified and averaged in determining the thickness of placental layers using ImageJ (NIH, Bethesda, MD, USA). Morphometric measurements were realized on light microscopy (Leica DMIRE 2, Leica Microsystems, Wetzlar, Germany). The proportion of each layer in the placenta was estimated by dividing the width of respective layer by the total mid-sagittal width of the placenta. Thickness of each zone was assessed using a superimposed graduated scale. For each placenta, data from all sections were averaged. For each litter, the individual data were averaged, so as the “n = 1” represented the value obtained for one litter.

### Statistical Analysis

All statistical analysis was performed using GraphPad Prism v5.0 for Windows (GraphPad Software, Inc, San Diego, USA). Outcome measures (continuous variables) between C and N-STZ groups were compared using one-way ANOVA followed by the Bonferroni posthoc test. Parameters for each fetus and placenta from the same litter are not independent measurement points. Therefore, an average was obtained for each litter in all measures. Thus, n = 1 represents averaged values in one litter and each individual female is considered as a single statistical unit. Spearman correlation coefficient was calculated to evaluate the correlation between the placenta weight and the birth weight. All data were tested for normality and homogeneity of variance. The differences were considered significant at p<0.05.

## Results

### N-STZ induces mild gestational hyperglycemia

**Figure 1:**
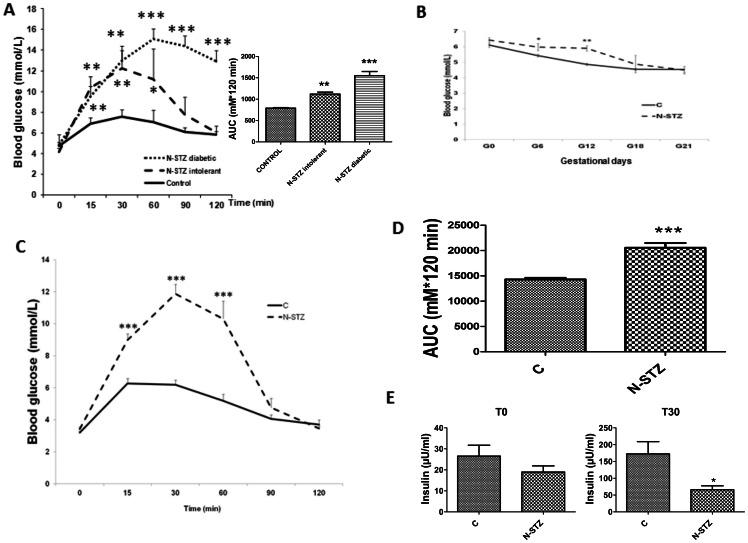
Oral glucose tolerance test (OGTT) in females before pregnancy and at GD19. (A) Blood glucose concentrations following an oral ingestion of glucose (2 mg/g body weight) in controls (n = 12) and N-STZ females (diabetic; n = 6 and intolerant; n = 14) before gestation. (B) Evolution of blood glucose concentration during the gestation in controls and N-STZ dams (C: n = 8, N-STZ: n = 10). (C) Blood glucose concentrations following an oral ingestion of glucose in pregnant controls and N-STZ females at GD19 (C: n = 8, N-STZ: n = 10). (D) AUC of blood glucose concentrations in response to OGTT in pregnant control and N-STZ females at GD19 (C: n = 8, N-STZ: n = 10). (E) Plasma insulin levels at fasting and at 30 min after glucose load in pregnant control and N-STZ females at GD19 (C: n = 8, N-STZ: n = 10). The effect of treatment was analyzed by one-way ANOVA followed by the Bonferroni posthoc test. Values represent mean ± SD. * p<0.05, ** p<0.01 indicate the significant differences between controls and N-STZ. C,control; N-STZ, nicotinamide-streptozotocin.

To determine the effect of N-STZ treatment on β-cell function under basal physiological conditions, we performed OGTT in non-pregnant females. Non-pregnant N-STZ females that have normal non-fasting glucose values (N-STZ: 7.29±1.42 mmol/l, *vs* C: 6.05±0.61 mmol/l) displayed impaired glucose tolerance before gestation (Figure 1A). N-STZ females that required insulin before mating had non-fasting glucose values significantly higher than control (N-STZ: 18.4±6.10 mmol/l, *vs* C: 6.05±0.61 mmol/l, p<0.001) and pronounced hyperglycemia in response to glucose (Figure 1A). These diabetic females were excluded from further analysis. The OGTT demonstrates that N-STZ females had a significantly greater area under the curve (AUC) compared to controls (Figure 1A). Non-fasting glucose values gradually decreased during pregnancy on both groups and no female in the N-STZ group became diabetic (Figure 1B). In order to determine if impaired glucose tolerance in N-STZ females was maintained during gestation, we performed OGTT in these animals at GD19 (Figure 1C). Fasted glucose levels were not significantly different between C and N-STZ females, but OGTT exhibit significant differences between the two groups and N-STZ dams showed greatest AUC (Figure 1D). To confirm that impaired glucose tolerance in N-STZ females is related to insulin deficiency, we measured plasma insulin levels during the OGTT. Although fasted insulin levels were not significantly different, insulin levels measured during the OGTT were significantly reduced in N-STZ females (Figure 1E). Therefore, the insulinogenic index value (It30 – It0/Gt30 -Gt0), which assesses the functionality of pancreatic β cells, is also decreased in N-STZ dams (N-STZ: 5.61±1.9 mU/mmol, *vs* C: 47.5±2.4 mU/mmol, p<0.001).

### Litter size and birth weight in control and N-STZ pregnancies

**Figure 2:**
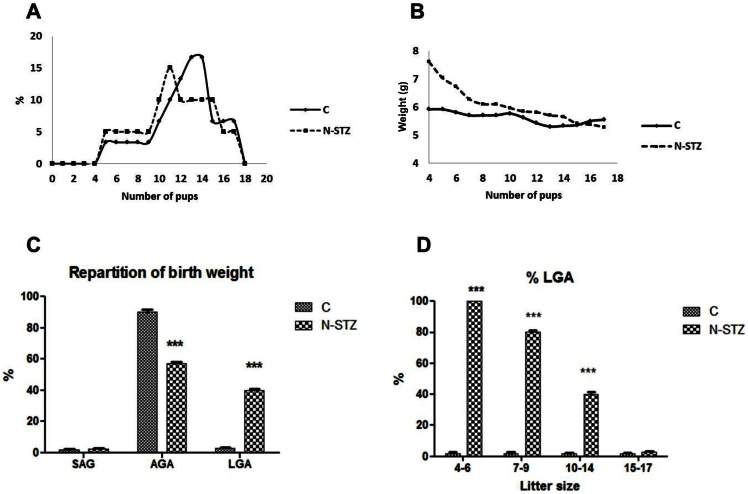
Litters size and birth weights in control and N-STZ pregnancies. (A) Repartition of litters in relation with the litter size. Results are expressed in percentage. (B) Body weight of pups at birth in relation with the litter size. (C) Classification of pups in relation with the birth weight in different categories: small for gestational age (SGA), appropriate for gestational age (AGA) and large for gestational age (LGA). (D) Percentage of LGA pups in relation with the litter size. The effect of treatment was analyzed by one-way ANOVA followed by the Bonferroni posthoc test. ***p<0.001 indicates the significant difference between controls and N-STZ. C, control; N-STZ, nicotinamide-streptozotocin.

The distribution of litter size in both control and N-STZ groups showed that more than 95% of litters had a number of pups comprised between 10 to 17 pups (Figure 2A). Litters with extreme ranges such as litters below 10 and above 17 fetuses, were not representative of the total population studied. Also we considered that these litters can be excluded from the analysis. There were significant differences in litter size between N-STZ and control pregnancies (N-STZ: 11.0±1.3 pups, *vs* C: 13.0±1.4 pups, p<0.05). Treatment with N-STZ did not affect the viability of the fetus and induced no fetal malformation. There is a long established inverse correlation between litter size and the weight of individual pups within a litter suggesting intrauterine competition for a limited supply of maternal resources [24]. We investigated whether there was evidence for influence of litter size by examining fetal weight in control and N-STZ litters with a different number of pups. As shown in figure 2, N-STZ pups were more overgrown in smaller litters compared with larger litters and there is an inverse correlation between litter size and body weight at birth (Figure 2B). Furthermore, the weight in control pups was similar irrespective of litter size (Figure 2B). These data could indicate that in the case of a normal pregnancy, the weight of the newborn is not influenced by the litter size whereas in N-STZ pregnancy there is a negative correlation between the newborn weight and litter size. Also, N-STZ pups from litters with 4 to 8 pups were 117% the weight of control (N-STZ: 6.86±0.011 g, *vs* C: 5.88±0.06 g, p<0.001), whereas the weight in N-STZ from litters of 15 pups or more was similar to control (N-STZ: 5.52±0.10 g, *vs* C: 5.65±0.09 g). These observations suggest that litter size may affect differentially the fetal growth in dams treated with N-STZ. We have classified the offspring born to control and N-STZ mothers according to their weight at birth. We first defined the distribution of birth weight of the newborns in the control population ( Figure S1). Fetuses that are large for gestational age (LGA) were considered as macrosomic if their birth weight was greater than 1.7 Standard Deviation (SD) of the curve of reference (birth weight > 90 ^th^ percentile) as previously reported [25]. Fetuses were considered as appropriate for gestational age (AGA) or normosomic if their birth weight was included in ±1.7 SD (birth weight between 11^th^ and 89 ^th^ percentile). Fetuses were considered as small for gestational age (SGA) if their birth weight was lower than 1.7 SD (birth weight <10 ^th^ percentile). The percentage of fetuses that are SGA was less than 2% and similar in both control and N-STZ dams (Figure 2C). By contrast, the percentage of fetuses that are LGA was significantly increased in N-STZ dams compared with controls (N-STZ: 40%, *vs* C: 2%, p<0.001). Thus, N-STZ administration before pregnancy was associated with fetal overgrowth rather than growth retardation. We also investigated whether the incidence of the macrosomia could be affected by the number of fetuses. Regardless the litter size, the percentage of newborns that are LGA or macrosomes in control group was less than 2% (Figure 2D). By contrast, this percentage increases in N-STZ group when the litter size is reduced (Figure 2D). Thus, the percentage of macrosomic newborns is on average 100% for litters with 4 to 6 pups, 80% for litters with 7 to 9 pups, 40% for litters with 10 to 14 pups and 2% for litters above 15 pups. Based on the incidence of macrosomia and the distribution of birth weight of the newborns that were different according to the litter size, we have identified the pregnancies with 10 to 14 pups as “normal” litters and those with 15 to 17 pups as “large” litters”.

### Body weight and metabolic parameters in N-STZ females

**Figure 3:**
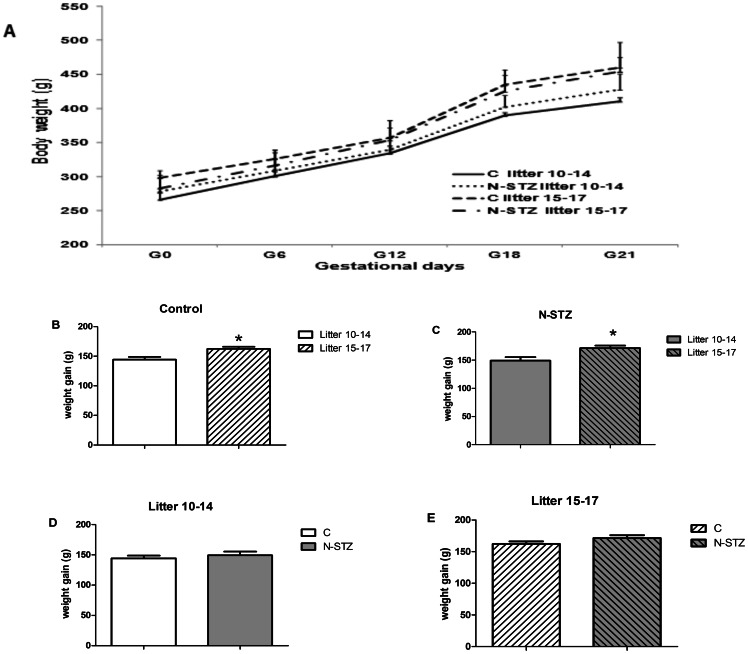
Maternal body weight in control and N-STZ pregnancies. (A) Evolution of maternal body weight during the gestation. (B, C) Maternal gain weight at G21 in relation with the litter size in control (B) and N-STZ (C) females. (D, E) Maternal gain weight at G21 in relation with the experimental group in litters 10–14 (D) and litters 15–17 (E). n = 4–6 females per group. The effects of the treatment and the litter size were analyzed by one-way ANOVA followed by the Bonferroni posthoc test. Values represent mean ± SD. * p<0.05 indicates the significant difference between the litters. C, control; N-STZ, nicotinamide-streptozotocin.

The OGTT in C and N-STZ pregnant dams did not exhibit a significant difference between the pregnancies with 10 to 14 pups and those with 15 to 17 pups (Figure S2). Irrespective of litter size, the maternal body weight on the day the vaginal plug was detected (GD0) was similar in all groups (Figure 3A). From the GD12, maternal weight varies differently depending on the litter size (Figure 3A). Weight gain is significantly increased among the females that carry 15 to 17 pups compared with females that have 10 to 14 pups, and at term, these females are heavier (Figure 3B and 3C). By contrast, there was no significant influence of N-STZ treatment on weight gain when the litter size was compared. N-STZ females have a weight gain similar to the controls when either litters with 10 to 14 pups or litters with 15 to 17 pups were compared (Figure 3D and 3E). However, the weight of hysterectomized females (weight of the dam minus the uterus, placentas and fetuses) was not different between all groups (data not shown). In control mothers, glucose, insulin, TG and NEFA levels were not significantly different between the two groups (Table 2). Thus, these metabolic parameters do not appear to be affected by the number of fetuses that are carried by the mother. N-STZ treatment reduced plasma NEFA concentrations in the mothers that have 10 to 14 fetuses but not among the mothers with 15 to 17 fetuses (Table 2). In contrast, plasma glucose, insulin and TG concentrations were not significantly different from controls.

**Table 2:** Metabolic parameters of pregnant dams.

Parameter	Normal litter		Large litter	
	C (n = 4)	N-STZ (n = 6)	C (n = 5)	N-STZ (n = 4)
Glucose (mmol/L)	4.73±0.38	4.45±0.38	4.40±0.33	4.65±0.33
Insulin (µU/ml)	36±11	28±10	26±10	20±9
Triglycéride (mg/dl)	179±55	205±34	271±61	200±30
NEFA (mmol/l)	0.43±0.03	0.24±0.06*, †	0.44±0.06	0.51±0.06

Number of dams per group appears in brackets. Values are mean. ± SEM. For maternal parameters, the effects of treatment (*) and litter size (†) were analyzed by one-way ANOVA with Bonferroni *posthoc* test. *p<0.05, (†). Metabolic parameters were measured in non fasted mothers at GD21.

### Fetal metabolic parameters

Plasmas from male and female newborns of the same litter were pooled and the parameters were measured separately. We found no difference in metabolic data between the sexes. Also, the values for males and females were averaged for each litter and are presented in Table 3. Circulating glucose, TG and NEFA levels in all groups were higher in maternal than in fetal blood. In contrast, plasma insulin levels were lower in the mothers compared to the fetuses. While the metabolic variables in maternal plasma did not differ with the number of pups in control pregnancies, the levels of insulin, TG and NEFA were lower in fetal blood from pregnancies with higher number of pups (Table 3). In pregnancies with 15–17 pups, metabolic variables did not differ significantly between control and N-STZ pups. In the pregnancies with 10–14 pups, we found that plasma glucose, TG and NEFA were significantly lower in N-STZ than in control pups (Table 3). Furthermore, insulin plasma levels were increased in N-STZ pups compared with controls (Table 3). In the N-STZ group, none of the fetal parameters measured can be correlated to placental weight or fetal growth. Thus, we did not compare the data on the plasma measurements from macrosomic pups to those from normosomic pups.

**Table 3:** Metabolic parameters of fetuses at birth.

Parameter	Normal litter		Large litter	
	C (n = 4)	N-STZ (n = 6)	C (n = 5)	N-STZ (n = 4)
Glucose (mmol/l)	3.79±0.11	3.52±0.11*	4.51±0.22	3.90±0.16
Insulin (µU/ml)	148.2±12	176.3±8*††	98±16†	67±9
Triglyceride (mg/dl)	116±17	72±7**	75±2†	93±10*
NEFA (mmol/l)	0.060±0.01	0.025±0.01**†	0.038±0.01†	0.039±0.01

The individual data were averaged for each litter, and n represents the number of litters per group. The effect of treatment (*) and litter size (†) were analyzed by one-way ANOVA with Bonferroni *post hoc* test. *p<0.05, **p<0.01 C; (†) p<0.05, (††) p<0.01.

### Feto-placental growth in N-STZ pregnancies

**Figure 4:**
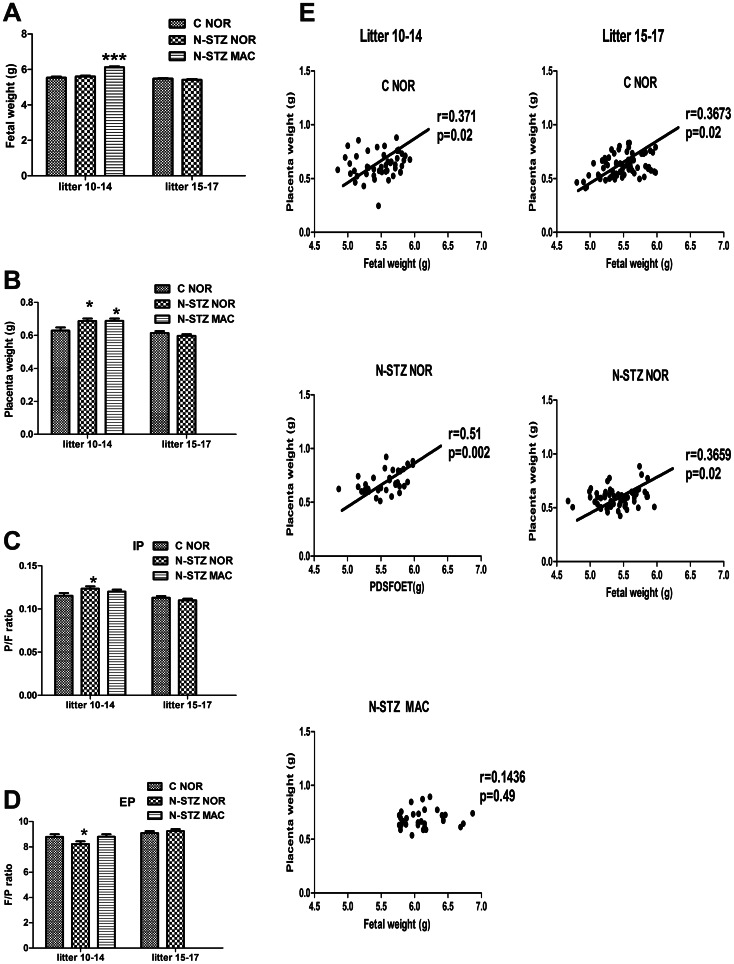
Fetal and placental growth in control and N-STZ pregnancies at GD21. (A) Body weight of pups. (B) Placenta weight. (C) Placenta on fetus ratio (P/F). (D) Fetus on placenta ratio (F/P). (E) Correlation between placenta and fetal weight. C-NOR, control normosomic; N-STZ NOR, nicotinamide-streptozotocin normosomic; N-STZ MAC, nicotinamide-streptozotocin macrosomic. n = 4–6 litters per group. The effect of treatment was analyzed by one-way ANOVA followed by the Bonferroni posthoc test. Values represent mean ± SD. * p<0.05 indicates the significant difference between controls and N-STZ. The coefficient of correlation was calculated by Spearman test.

N-STZ newborns from litters 10–14 were heavier than controls (N-STZ: 5.84±0.10 g, *vs* C: 5.56±0.10 g, p<0.01) and the percentage of macrosomic pups was increased significantly (N-STZ, n = 73: 40% in males (14/35) and 47% in females (18/38), *vs* C, n = 49: 3% in males (1/32) and 0% in females (0/17), p<0.001). In contrast, the body weight of newborns from litters 15–17 was not different between the two groups (N-STZ: 5.52±0.10 g, *vs* C: 5.65±0.08 g) and the percentage of macrosomic pups was less than 5% in both groups. The birth weight was significantly increased in N-STZ macrosomic pups relative to the N-STZ normosomic and control newborns (Figure 4A). The placental weight (Figure 4B) was also higher among the N-STZ dams that had 10 to 14 pups compared to the placentas from control females (N-STZ: 0.743±0.06 g, *vs* C: 0.649±0.07 g, p<0.05). However, there is no significant difference in the N-STZ placentas between macrosomic and normosomic pups. In contrast, the weight of the placentas in pregnancies with 15 to 17 pups was not different between control and N-STZ pregnancies (N-STZ: 0.608±0.03 g, *vs* C: 0.639±0.04 g). In spite of the fetal overgrowth, the placental weight of macrosomic pups was not significantly different from that of normosomic pups either in control or in N-STZ pregnancies (Figure 4B). The ratio of placental to fetal weight (P/F ratio) as well as the fetal to placental weight ratio (F/P ratio) was also similar in the macrosomic pups compared to the normosomic pups (Figure 4C and 4D respectively). However, placental weight was positively correlated with birth weight within each of the normosomic groups but not among the macrosomic pups (Figure 4E). This indicates the existence of a non-harmonious development of the fetus and its placenta in the case of macrosomia unlike to what is observed in the control pregnancies.

### Placental morphology in N-STZ pregnancies

**Figure 5:**
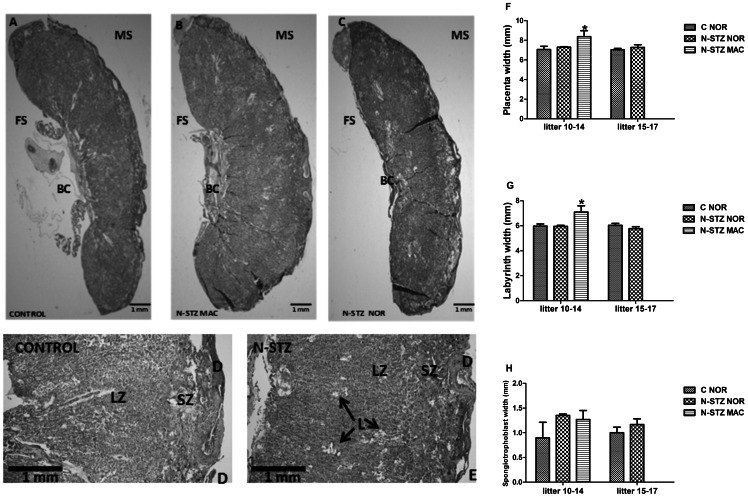
Morphological analyses of control and N-STZ placentas at GD21. (A, D) Histological images of control placentas. (B–E) Histological images of N-STZ placentas. Paraffin-embedded sections were stained with hematoxylin-eosin-safran for morphologic analyses under light microscopy. (F–H) Absolute width of the total placenta (F), labyrinthine layer (G) and spongiotrophoblast layer (H). The effect of treatment was analyzed by one-way ANOVA followed by the Bonferroni posthoc test. Values represent mean ± SD (n = 3–4 animals per group). *p<0.05 indicates the significant difference between controls and N-STZ. BC, blood cord; D, decidua; FS, fetal side; LZ, labyrinthine layer; L, lacunae; MS, maternal side; SZ, spongiotrophoblast layer. C, control; N-STZ, nicotinamide-streptozotocin; C-NOR, control normosomic; N-STZ NOR, nicotinamide-streptozotocin normosomic; N-STZ MAC, nicotinamide-streptozotocin macrosomic.

Histological analysis showed the presence of the three major layers in N-STZ placentas (Figures 5A, 5B and 5C): the decidua and the spongiotrophoblast that represent the junctional zone of the placenta which is located at the maternal face, and the labyrinth that is situated on the fetal side near to the chorionic plate. However, the presence of lacunae within the labyrinth is more frequent in N-STZ than in controls (N-STZ: 38%, *vs* C: 22%), but not restricted to the N-STZ placentas of the macrosomic pups (Figures 5D and 5E). Quantitative analyses revealed that the size of the placenta as well as the areas occupied by spongiotrophoblast and labyrinthine layers were similar in control placentas from normal and large litters (Figure 5F–5H). Histological data showed an overall higher size of N-STZ placentas from macrosomic pups compared to the N-STZ placentas from normosomic pups and those of controls (Figures 5A–5C and 5F). While the labyrinthine zone was increased in the N-STZ placentas from macrosomic pups, the area occupied by spongiotrophoblast layer was not significantly altered (Figures 5G and 5H). Furthermore, all measurements that are performed in the N-STZ placentas of normosomic pups did not differ from those of controls (Figures 5F, 5G and 5H).

### Placental gene expression in N-STZ pregnancies

We performed RT-qPCR analysis to evaluate the effects of litter size and N-STZ treatment on gene expression in the placenta samples. First, we compared gene expression in control placentas between pregnancies with normal (10–14 pups) and large (15–17 pups) litters. Second, in order to determine whether some genes were associated to fetal overgrowth, we compared the N-STZ placentas from macrosomic pups to the placentas of control and N-STZ normosomic pups. We obtained expression data for five groups: control and N-STZ placentas of normosomic pups from normal and large litters (C NOR, N-STZ NOR) and N-STZ placentas of macrosomic pups from normal litters (N-STZ MAC). Placenta samples were analyzed separately and the individual data were averaged according to experimental group for each litter.

**Figure 6:**
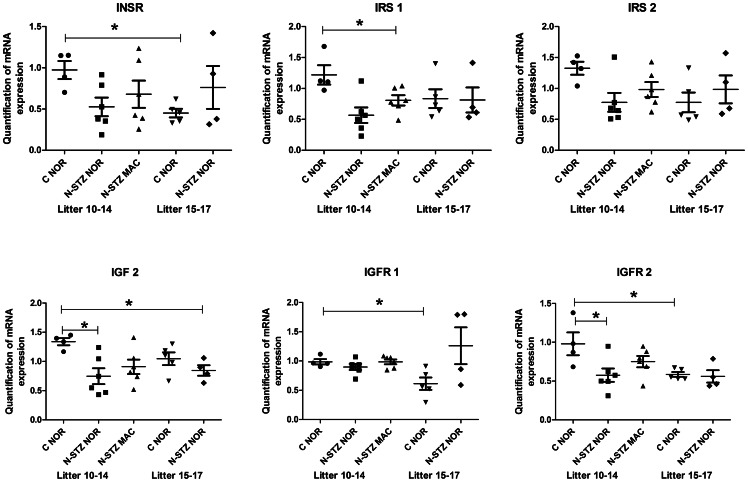
Normalized real-time PCR transcripts of insulin/IGFs system genes in control and N-STZ placentas at GD21. Each symbol represents the mean of placental gene expression from pups within a single litter (n = 4–6 litters per group). Solid line indicates median values for all of the litters. The effects of treatment and litter size were analyzed by one-way ANOVA followed by the Bonferroni posthoc test. Values represent mean ± SD. Horizontal line indicates the comparison between the two groups. * p<0.05 shows the significant difference between groups. C-NOR, control normosomic; N-STZ NOR, nicotinamide-streptozotocin normosomic; N-STZ MAC, nicotinamide-streptozotocin macrosomic.

**Figure 7:**
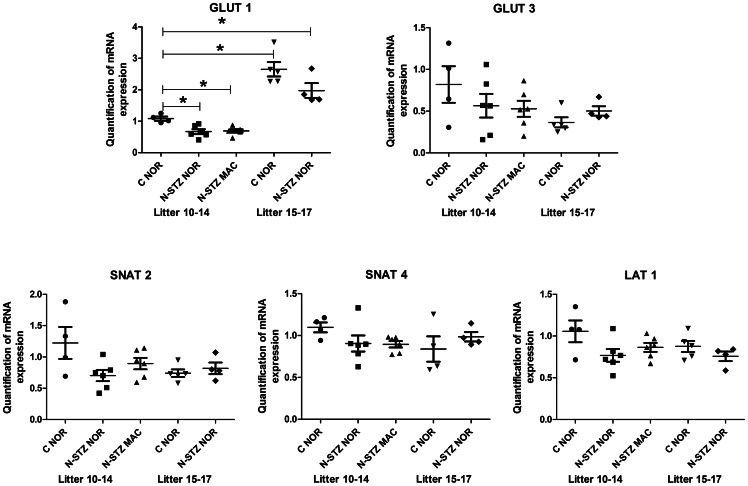
Normalized real-time PCR transcripts of nutrient transporter genes in control and N-STZ placentas at GD21. Each symbol represents the mean of placental gene expression from pups within a single litter (n = 4–6 litters per group). Solid line indicates median values for all of the litters. The effects of treatment and litter size were analyzed by one-way ANOVA followed by the Bonferroni posthoc test. Values represent mean ± SD. Horizontal line indicates the comparison between the two groups. * p<0.05 shows the significant difference between groups. C-NOR, control normosomic; N-STZ NOR, nicotinamide-streptozotocin normosomic; N-STZ MAC, nicotinamide-streptozotocin macrosomic.

**Figure 8:**
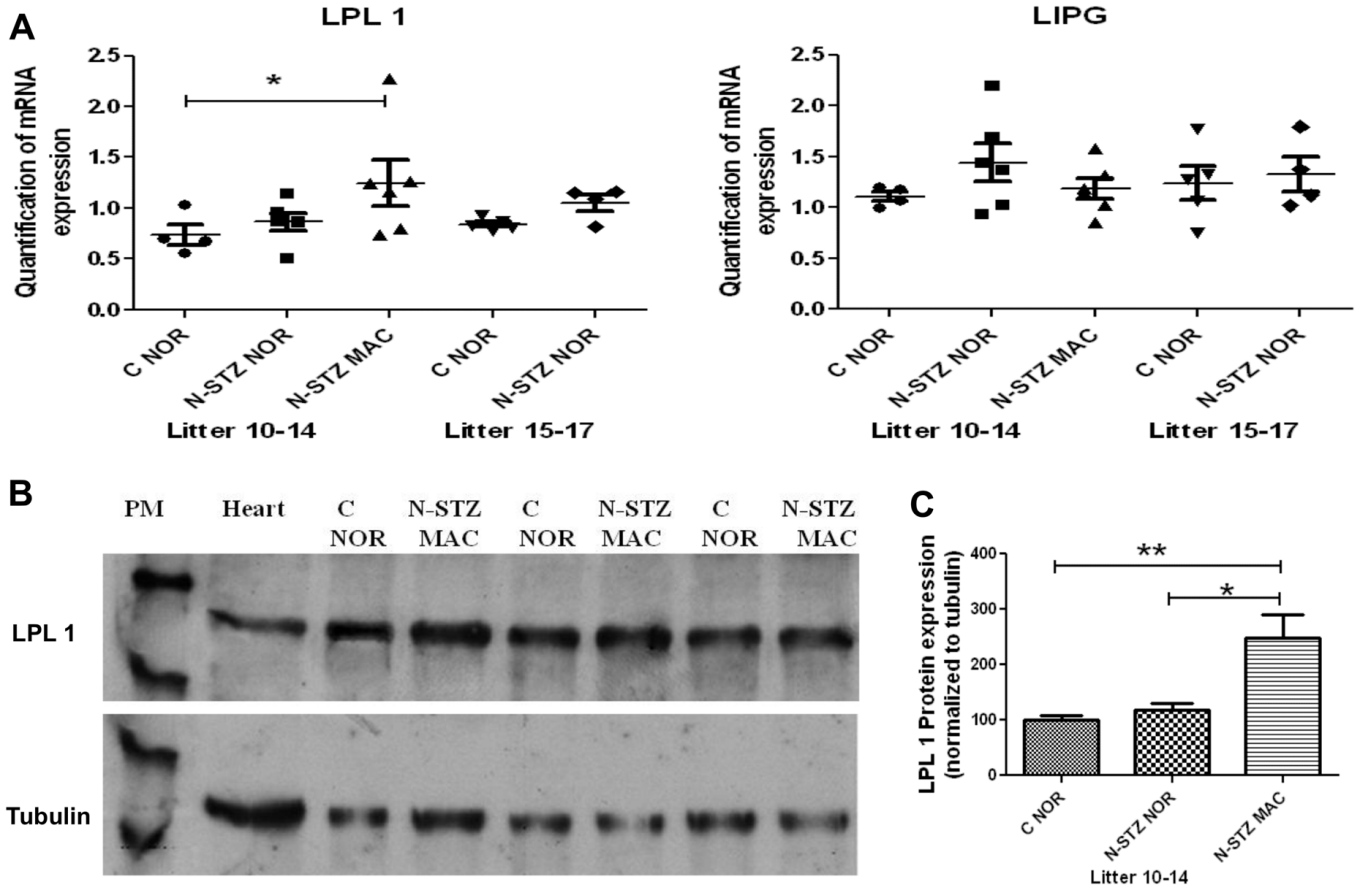
Lipoprotein lipase expression in the control and N-STZ placentas at GD21. (A) Normalized real-time PCR transcripts of LPL1 and LIPG genes. Each symbol represents the mean of placental gene expression from pups within a single litter (n = 4–6 litters per group). Solid line indicates median values for all of the litters. (B) Representative western blots and (C) densitometry values for LPL1 protein expression in control and N-STZ placentas. Representative blots show expression within three paired litters (n = 4–6 litters). Rat heart was used as a positive control. The effects of treatment and litter size were analyzed by one-way ANOVA followed by the Bonferroni posthoc test. Values represent mean ± SD. Horizontal lines indicates the comparison between the two groups. * p<0.05 and **p<0.01 show the significant differences between groups. C-NOR, control normosomic; N-STZ NOR, nicotinamide-streptozotocin.normosomic; N-STZ MAC, nicotinamide-streptozotocin macrosomic.

We selected 19 genes for their roles in vasculogenesis and angiogenesis, differentiation and proliferation, metabolism and nutrients transport and likely to have profound effects on fetal growth. RT-qPCR analysis showed no detectable expression for the glucose transporter isoform *Slc2a4*/GLUT4 and LIPE genes, and a very low expression for the amino acid transporter isoform *Slc38a1*/SNAT1 gene in both control and N-STZ placentas (data not shown). For some of these genes, we found that expression levels were different among pregnancies with 15–17 pups (large litter) compared to those with 10–14 pups (normal litter). The results revealed that the expression of INSR, IGFR1, IGFR2 were significantly down regulated in the placentas of control pups (C NOR) from mothers that have large litters compared to those carrying normal litters (Figure 6). Glucose transporter genes were also modulated in control placentas from large litters compared to normal litters (Figure 7). Particularly, glucose transporter *Slc2a1*/GLUT1 expression levels were significantly increased whereas those of *Slc2a3*/GLUT3 were not modified (Figure 7). In contrast, the expression of amino acid transporter genes was not significantly different in the control placentas from pregnancies with large litters compared to normal litters (Figure 7). When we analyzed the expression of genes in the N-STZ placentas, we found no differences compared with controls (Figures 6 and 7). Among the genes involved in the placental transfer of lipids, we focused our analysis on those related to the transport of VLDL, namely triglycerides lipases LPL1 and LIPG, and VLDLR genes. We showed that the expression of LPL1, LIPG (Figure 8) and VLDLR (data not shown) genes were not different between control placentas from large litters in comparison to control placentas from normal litters and their expression were not modified in N-STZ placentas. These data suggest that several genes, especially genes of the growth factors and the gene GLUT1 may be modulated in the placentas from control pregnancies when the number of fetuses is higher than 15. The maternal mild hyperglycemia in N-STZ pregnancies did not change further the expression of these genes. Finally, we investigated the placental genes expression in control and N-STZ pregnancies with normal litters (10–14 pups). For this purpose, we compared the data between control (C NOR) and N-STZ (N-STZ NOR or N-STZ MAC) placentas. Notably, we determinate whether there was a differential gene expression between the placentas of macrosomic (N-STZ MAC) and normosomic (C NOR and N-STZ NOR) pups. We found that the expression of IGF2 and IGFR2 genes were significantly down regulated in the N-STZ placentas of normosomic pups, but not in the placentas of macrosomic pups in comparison to controls (Figure 6). However, the expression of IRS1 gene was significantly reduced in the N-STZ placentas of macrosomic pups compared to controls whereas it does not in normosomic ones (Figure 6). Placental expression of the glucose transporter genes was also down regulated in N-STZ pregnancies. Notably, the expression of *Slc2a1*/GLUT1 gene was significantly decreased in the N-STZ placentas of normosomic pups but also in those of macrosomic pups, whereas the expression of *Slc2a3*/GLUT 3 gene was not different from controls (Figure 7). In contrast, the expression of amino acid transporter genes was not modified in the N-STZ placentas from normosomic or macrosomic pups compared to controls (Figure 7). These data suggested that in the pregnancies with normal litters, unlike to the pregnancies with large litters, moderate hyperglycemia during gestation can modulate the expression of IGFs and GLUT1 genes in the placenta, regardless of normosomic or macrosomic phenotype. We found that LPL1 was differentially expressed in the N-STZ placentas of macrosomic pups. Notably, the expression of LPL1 gene was significantly increased in the N-STZ placentas of macrosomic pups but not in the N-STZ placentas of normosomic pups compared to the control placentas (Figure 8A). However, the expression levels of the LPL1 gene in the macrosomic placentas were not significantly different from those of normotrophes (Figure 8A). In contrast, the expression of LIPG (Figure 8A) and VLDLR (data not shown) genes in the N-STZ placentas of both normosomic and macrosomic pups was not different from controls. Finally, the expression of angiogenic genes PLGF and VEGF was not modulated neither in control pregnancies with normal or large litters nor in N-STZ pregnancies (data not shown).

### LPL1 protein expression

Among the genes studied, we have previously identified LPL1 gene as the only gene that was specifically up regulated in the placentas of macrosomic pups. Therefore, we investigated whether variations in the LPL1 protein expression were also observed in macrosomic placentas, in comparison to N-STZ placentas of normosomic pups or to control ones. Western blot analysis showed that LPL1 protein was expressed as a single band of approximately 56 kDa, corresponding to the mature form of the enzyme, both in control and N-STZ placentas (Figure 8B). LPL1 protein expression was significantly increased in the N-STZ placentas of macrosomic pups compared with those of normosomic pups or controls (Figure 8C).

## Discussion

In this paper, we describe a relevant model in rat in which concomitant injection of streptozotocin and nicotinamide impairs glucose tolerance in the mother, but did not induces diabetes. We report that N-STZ pregnancies increased the incidence of fetal overgrowth according to the number of pups that was present within the litter. Fetal growth was associated with increased placental weight and labyrinth layer development. Furthermore, we identified LPL1 gene and protein whose expression was up-regulated in placentas from macrosomic pups. We report here that genes expression was also significantly altered in the placentas from control pregnancies with larger litters.

Most experimental models of diabetes induced by STZ administration cause insulin deficiency and permanent hyperglycemia, and are related to moderate and/or severe T1DM. Furthermore, STZ-induced diabetes during pregnancies was often associated with congenital malformations and leads to reduction of intrauterine growth at later stages [16]. The difficulties to obtain moderate hyperglycemia in rodents treated with low doses of STZ may be related to the interaction of polygenic and nutritional factors that lead to different responses to the diabetogenic agent in beta-cells. Indeed, maternal diet has been shown to influence the extent of reduced fetal size in streptozotocin-induced diabetes mouse model [26], [27]. To circumvent this question, we have chosen another strategy to induce the partial destruction of the beta-cells mass. Administration of nicotinamide plus STZ to virgin animals before mating leads to euglycemia in 14 out of 20 females (70%) despite a relative insulin deficiency. Few females (6 out of 20 females) developed overt diabetes that requires insulin therapy and were excluded from this study. N-STZ females displayed impaired glucose tolerance before mating as well as during pregnancy. Furthermore, N-STZ dams have decreased plasma insulin levels in response to glucose load, indicating that insulin production and secretion are not sufficient to maintain glucose homeostasis. Therefore, the glycemic response in N-STZ rats is closer to that found in pregnant women with mild gestational hyperglycemia than in women with gestational diabetes [4]. In contrast with models of diabetes that were usually described in the literature, the N-STZ treatment had no significant effect on the successful mating as well as on the embryo malformations, and resulted in fetal overgrowth.

We report also that the occurrence of macrosomic newborns depends on the number of fetuses that are carried by the mother. The incidence of LGA newborns reached 40% in N-STZ dams that have 10 to 14 pups, whereas in N-STZ dams that have above 15 pups the macrosomia affects only 5% of the newborns. This observation appears consistent with clinical studies that show a decrease in the incidence of macrosomia in twin pregnancies complicated with diabetes compared with singleton pregnancies [28]. These data could be indicative of the limitations of the mother in supporting a pregnancy with large fetuses when their number becomes too important. In this case, it could be an adaptive mechanism whose role is decisive for pregnancy success. In the context of pregnancies complicated with diabetes, not only glucose but also maternal lipids may contribute to the risk of having LGA newborns [29], [30]. Clinical studies showed an enhanced insulin resistance in women with gestational diabetes that contributes to significantly increase the lipids concentrations, in particular triglycerides and NEFA, in late gestation [31]–[33]. Furthermore, the circulating triglycerides and NEFA concentrations were positively correlated with neonatal weight at birth [33], [34]. There are also studies where no change in plasma lipid levels was found in GDM compared to control pregnant women [29]. We show that N-STZ dams who have the highest incidence of macrosomic newborns also exhibit disturbances in lipid concentrations, in addition to impaired glucose tolerance. In these animals, maternal plasma levels of NEFA were significantly decreased whereas triglycerides concentrations were slightly increased. It is possible that the combined contribution of moderate insulin deficiency and insulin-resistant condition taking place during late pregnancy may promote the lipolysis and the release of NEFA into the circulation. The latter are then directed to the liver where they are re-esterified for the synthesis of triglycerides and the production of VLDL. The decrease in plasma NEFA could correspond to an enhanced removal from the circulation as consequence of improved VLDL production by the liver and increased placental transfer of lipids. Contrary to pregnant women with GDM that are overweight or obese, we showed that N-STZ dams had a normal weight before the pregnancy and displayed a gain weight similar to controls during gestation. The possibility exists that the degree of metabolic control may be different in N-STZ females, which would explain, in part, the reasons for this discrepancy.

Triglycerides represent the primary component of lipid stores in fetuses, and are synthesized from circulating NEFA or from lipogenic precursors such as glucose. The concentrations of triglycerides and NEFA were lower in the plasma of newborns of the N-STZ dams that have given birth to macrosomes. Fetal hyperinsulinism, which was also present in these pups is known to promote fat accretion and may be responsible for the reduced fetal lipid levels. Additionally, maternal moderate hyperglycemia may induce a greater placental transfer of glucose to the fetus and, hence, an increased availability of lipogenic substrates. However, this relationship was not found in newborns of N-STZ dams having only normosomic pups, suggesting that beta – cells responsiveness to glucose was impaired and/or lipids utilization was reduced. Further investigations could be conducted to determine whether the responses are different between macrosomic and normosomic newborns.

Fetal growth depends on availability of nutrients derived from maternal circulation and transferred through the placenta. It is thus conceivable that changes in the placental function would impact fetal growth. Here, we report that placental growth and gene expression were affected in N-STZ pregnancies that have given birth to macrosomic newborns. We showed that placental weight, and to a lesser extent, placental index (placenta weight/birth weight ratio) and placental efficiency (birth weight/placenta weight ratio) were altered in macrosomic newborns but also in normosomic newborns. However, at term, placental and fetal weights are strongly correlated among the normosomic newborns from N-STZ or control dams. In contrast, this linear relationship does not exist in macrosomic pups, which is indicative of the disharmonious development of the fetus and its placenta.

Furthermore, histological analysis revealed the overall higher size and enlarged labyrinthine layer in the macrosomic placentas compared with normosomic placentas, whereas the spongiotrophoblast size was not significantly altered. Reduced labyrinth size and impaired spongiotrophoblast differentiation have been recently reported in diabetic mouse placentas [35], [36]. The authors suggested that these alterations which appear at temporally distinct phases during gestation could contribute to reduced fetal growth described in this animal model of diabetic pregnancy [27], [36]. Our data do not unequivocally demonstrate that the rise in labyrinthine zone is responsible for fetal overgrowth, because the possibility that changes occur at early stages of gestation has not been evaluated in the current study.

The molecular mechanisms underlying fetal overgrowth in pregnancies complicated with mild hyperglycemia had not yet been investigated. This is the first study that explores the expression of genes known to be involved in the placental development and fetal growth in a model of MGH. We report that in the N-STZ pregnancies with a higher risk to have macrosomic newborns, the expression of IGF2, IGFR2 and IRS1 genes in the placentas was down regulated compared to controls. While the placentas from macrosomic pups have a reduced IRS1 gene expression, those of normotrophes were associated with a down regulation of IGF2 and IGFR2 genes. Insulin/IGFs system constitutes a major determinant for the growth of the placenta and has been shown to play a significant role in the placental adaptations in response to the nutritional challenges in several rodent species [37], [38]. In accordance, we show that the placental weight was higher in macrosomic newborns but also in normotrophes ones. We also found that the expression of GLUT1 gene is decreased in the N-STZ placentas of macrosomic and normosomic pups. The effects of maternal diabetes on the placental expression of glucose transporters were often contradictory, and not linked to fetal growth [39]–[43]. The reasons for these discrepancies may be explained by the temporal expression of glucose transporters isoforms during the gestation facing to the gestational period at which the analyses were conducted [44]. For example, high fat diet in mice before and during the pregnancy increases transplacental transport of glucose and causes up regulation of protein expression of GLUT1 in the microvillous plasma membranes from placentas at embryonic day 18.5 [43]. Furthermore, these changes were associated with fetal overgrowth at this stage of development suggesting that they could be a cause of altered fetal growth [43]. However, the high fat diet induced maternal obesity that was correlated with increased circulating leptin and decreased serum adiponectin concentrations, but normal glucose tolerance [43]. Thereby, maternal metabolic profile was strongly different from that of the N-STZ mothers and could expose the feto-placental unit to a hormonal environment distinct because the leptin and adiponectin have been reported to regulate the transfer of nutriments through the placenta [45]. Taken together, our data demonstrate that moderate hyperglycemia and/or glucose intolerance during pregnancy modulate the expression of several genes involved in the placental development and glucose transport without necessarily having an impact on weight at birth. Interestingly, these genes are also affected in the placentas from control dams that give birth to large litters. Contrary to pregnancies giving birth to normal litters, maternal hyperglycemia had no additional effect on their expression level. The reasons of these modifications are not understood, but they may correspond to functional adaptations if the number of fetuses exceeds the capacity of the uterus and/or maternal resources to ensure the success of the pregnancy. Again, these changes were independent of maternal blood glucose (normoglycemia *vs* moderate hyperglycemia) and the phenotype of newborns at birth (i.e macrosomic *vs* normosomic). We have identified LPL1 whose expression is specifically modulated in the placentas from macrosomic newborns, whereas the expression of other members of triglycerides lipases was unchanged. In particular, we showed that gene expression as well as that of the protein was significantly increased in N-STZ placentas from macrosomic pups. In mouse placentas, LPL1 was strongly expressed in the labyrinthine layer [46] where it participates to TGs-rich lipoproteins hydrolysis, contributing thus to the placental transport of NEFA. The up-regulation of LPL1 in macrosomic placentas could be indicative of enhanced transplacental transfer of lipids since the lipid metabolism of their mothers, as mentioned above, was also disrupted. It would be required to investigate the distribution of LPL1 at the cellular level (maternal or fetal side) to gain more insight into its physiological roles on lipids transfer in the placenta. Lipoprotein metabolism and lipids transports have been reported to be altered in models [47]–[49] and in women pregnancies complicated with diabetes [29], [50]. Recent studies have identified the LPL1 gene as diabetes-responsive gene under conditions of maternal diabetes [26], [36]. The authors show that reduced levels of LPL1 expression in diabetic placentas was associated with concomitant impaired fetal growth. Our data do not conclude unequivocally that the up-regulated gene expression of LPL1 in the placenta is the cause of fetal growth, because the measurements were realized at birth. Further studies must be conducted at early stages of development to address to this question.

Taken together, our results demonstrate that placental gene expression was modulated by gestational conditions that might disrupt the fetal growth. We consider that N-STZ model offers the opportunity to determinate whether maternal lipids metabolism and placental lipids transport may contribute to the fetal overgrowth. Because mild gestational hyperglycemia is also associated with perinatal anomalies, the placental alterations we find in N-STZ pregnancies may have implications for developmental programming of metabolic dysfunctions in later life.

## Supporting Information

Figure S1 **Macrosomia is defined as mean ± 1.7 SD of control body weight and expressed as percentage for male (A) and female (B).**
(TIF)

Figure S2 **Blood glucose concentrations following an oral ingestion of glucose in pregnant controls and N-STZ females (n = 4–6) at GD19 and AUC of blood glucose concentrations.** The effect of treatment was analyzed by one-way ANOVA followed by the Bonferroni posthoc test. Values represent mean ± SD. * p<0.05. **p<0.01 show the significant differences between controls and N-STZ.
(TIF)
