# Supplementary figures and images for: Mild Gestational Hyperglycemia in Rat Induces Fetal Overgrowth and Modulates Placental Growth Factors and Nutrient Transporters Expression
Source: PLoS One. 2013 May 14;8(5):e64251. doi: 10.1371/journal.pone.0064251 (PMC3653871; doi:10.1371/journal.pone.0064251)

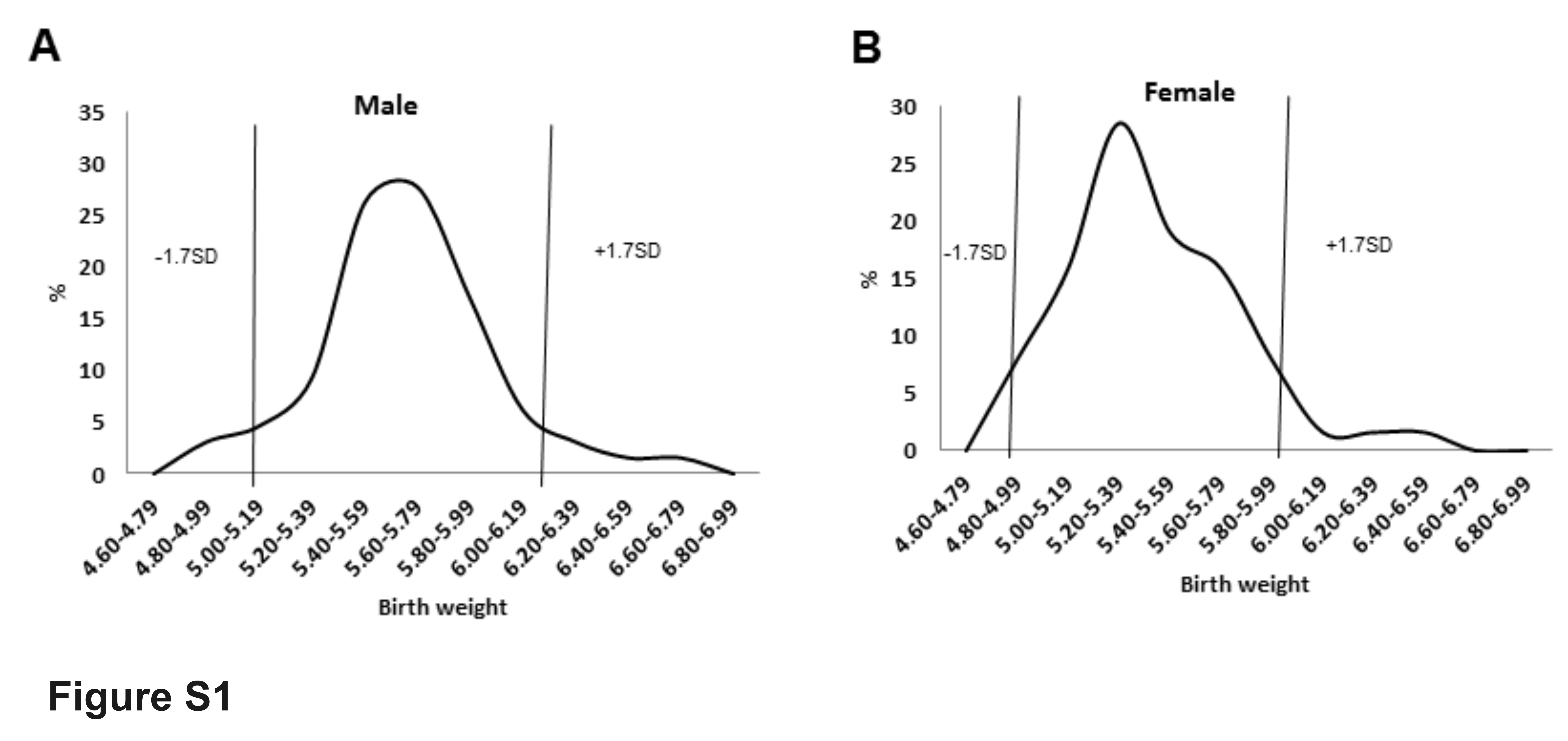

Supplement: Figure S1 — Macrosomia is defined as mean ± 1.7 SD of control body weight and expressed as percentage for male (A) and female (B). (TIF) [file pone.0064251.s001.tif]

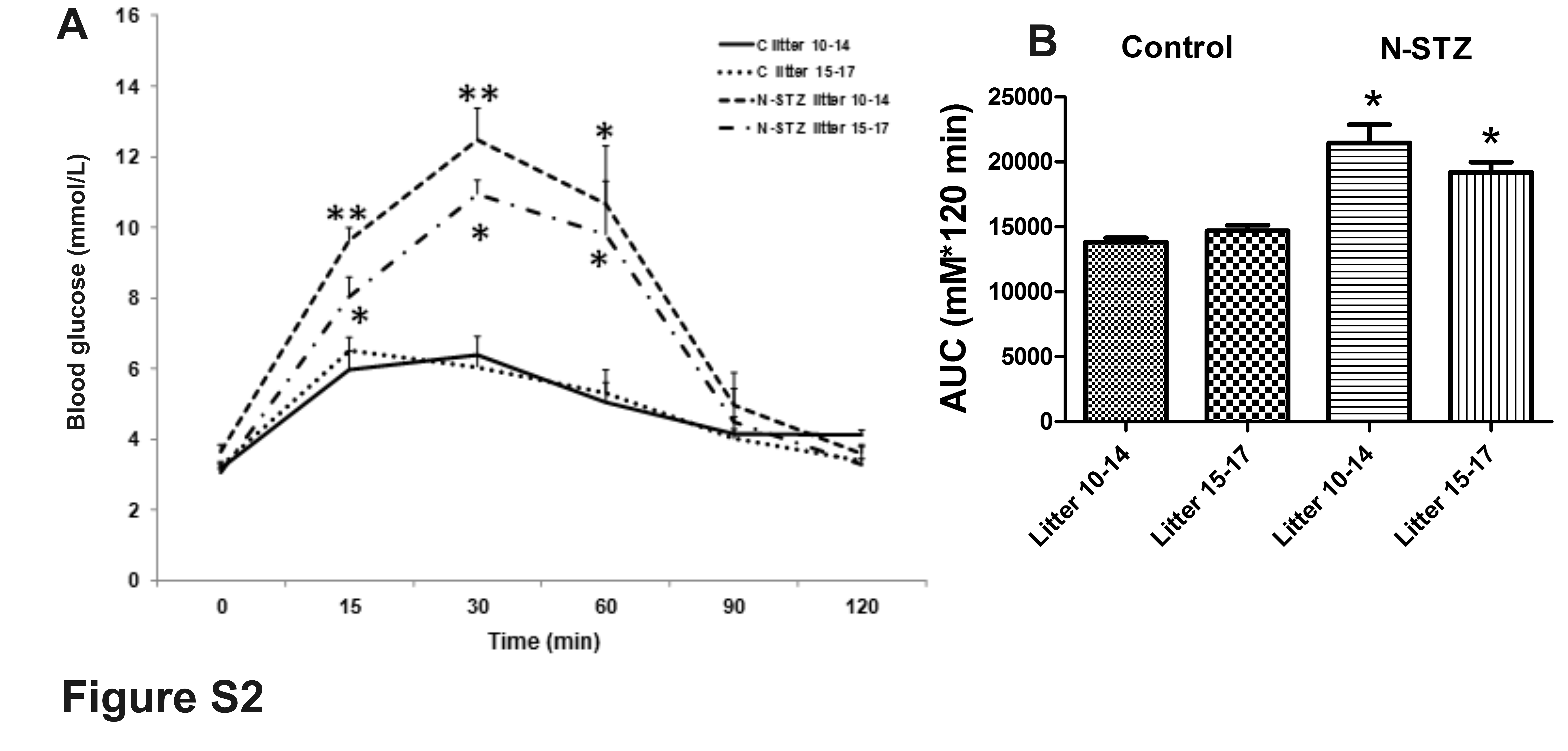

Supplement: Figure S2 — Blood glucose concentrations following an oral ingestion of glucose in pregnant controls and N-STZ females (n = 4–6) at GD19 and AUC of blood glucose concentrations. The effect of treatment was analyzed by one-way ANOVA followed by the Bonferroni posthoc test. Values represent mean ± SD. * p<0.05. **p<0.01 show the significant differences between controls and N-STZ. (TIF) [file pone.0064251.s002.tif]
